# Negative serum (1,3) -β-D-glucan has a low power to exclude *Pneumocystis jirovecii* pneumonia (PJP) in HIV-uninfected patients with positive qPCR

**DOI:** 10.1186/s12941-023-00650-7

**Published:** 2023-11-20

**Authors:** Yuan Huang, Jie Yi, Jing-jing Song, Li-jun Du, Xiao-meng Li, Lin-lin Cheng, Song-xin Yan, Hao-long Li, Yong-mei Liu, Hao-ting Zhan, Ya-ling Dou, Yong-zhe Li

**Affiliations:** 1 Department of Clinical Laboratory, Peking Union Medical College Hospital, Dongcheng District, Beijing, 100730 China; 2 Department of Clinical Laboratory, The Maternal and Child Health Hospital Affiliated to Anhui Medical University, Hefei, Anhui 230001 China; 3 Department of Clinical Laboratory, Nanchong Central Hospital, the Second Clinical Medical College, North Sichuan Medical College, Nanchong, Sichuan Province 637000 China

**Keywords:** *Pneumocystis Jirovecii* pneumonia (PJP), *Pneumocystis Jirovecii* colonization, (1,3)-beta-D glucan (BDG), HIV-uninfected patients, Differential diagnosis, Biomarkers

## Abstract

**Objective:**

The current study evaluated the diagnostic performance of serum (1,3)-beta-D Glucan (BDG) in differentiating PJP from *P. jirovecii*-colonization in HIV-uninfected patients with *P. jirovecii* PCR-positive results.

**Methods:**

This was a single-center retrospective study between 2019 and 2021. The diagnosis of PJP was based on the following criteria: detection of *P. jirovecii* in sputum or BAL specimen by qPCR or microscopy; Meet at least two of the three criteria: (1) have respiratory symptoms of cough and/or dyspnea, hypoxia; (2) typical radiological picture findings; (3) receiving a complete PJP treatment. After exclusion, the participants were divided into derivation and validation cohorts. The derivation cohort defined the cut-off value of serum BDG. Then, it was verified using the validation cohort.

**Results:**

Two hundred and thirteen HIV-uninfected patients were enrolled, with 159 PJP and 54 *P. jirovecii*-colonized patients. BDG had outstanding specificity, LR, and PPV for PJP in both the derivation (90.00%, 8.900, and 96.43%) and the validation (91.67%, 9.176, and 96.30%) cohorts at ≥ 117.7 pg/mL. However, it had lower sensitivity and NPV in the derivation cohort (89.01% and 72.97%), which was even lower in the validation cohort (76.47% and 57.89%). Of note, BDG ≥ 117.7 pg/mL has insufficient diagnostic efficacy for PJP in patients with lung cancer, interstitial lung disease (ILD) and nephrotic syndrome. And although lymphocytes, B cells, and CD4^+^ T cells in PJP patients were significantly lower than those in *P. jirovecii*-colonized patients, the number and proportion of peripheral blood lymphocytes did not affect the diagnostic efficacy of serum BDG.

**Conclusions:**

Serum BDG ≥ 117.7 pg/mL could effectively distinguish *P. jirovecii*-colonization from infection in qPCR-positive HIV-uninfected patients with infectious diseases, solid tumors (excluding lung cancer), autoimmune or inflammatory disorders, and hematological malignancies. Of note, for patients with lung cancer, ILD, and nephrotic diseases, PJP should be cautiously excluded at BDG < 117.7 pg/mL.

**Supplementary Information:**

The online version contains supplementary material available at 10.1186/s12941-023-00650-7.

## Introduction

*Pneumocystis jirovecii* (formerly *Pneumocystis carinii*) pneumonia (PJP), is a life-threatening fungal infection predominantly observed in immunocompromised individuals. HIV-uninfected populations have underlying risk factors for immunodeficiency, including hematological malignancies, stem-cell or solid-organ transplants, autoimmune diseases, and solid cancers. They are at increased risk for developing PJP [1]. More atypical clinical manifestations, faster disease progression, and a higher risk of respiratory failure than in HIV-infected patients characterizes PJP in HIV-uninfected patients [2, 3]. With the rising morbidity and mortality of PJP, timely and accurate diagnosis is essential. Therefore, it requires typical clinical features and microbiological confirmation [4].

Current methods for the etiological diagnosis of PJP depend on *P. jirovecii* detection from induced sputum and/or bronchoalveolar lavage fluid [5] using cytological staining and conventional or quantitative PCR [6]. However, each diagnostic method has its limitations. The microscopic examination of respiratory specimens has low sensitivity [7]. In contrast, conventional PCR enhances diagnostic sensitivity but cannot distinguish asymptomatic colonization, which was defined as molecular detection without positive clinical signs of PJP [8, 9]. Although quantitative PCR demonstrates the potential for differentiating colonization from infection, it lacks a unified cut-off value and demands clinical validation [10].

*P. jirovecii* colonization is an essential clinical phenomenon in immunocompromised HIV-uninfected patients [11], young children [12, 13], and even in immunocompetent subjects [14, 15]. Therefore, clinically distinguishing *P. jirovecii* colonization from PJP is a great challenge in patients with significant medical complexity and a high possibility of *P. jirovecii* colonization.

Serum (1,3)-beta-D Glucan (BDG) is an antigenic cell wall component of many fungi, including *P. jirovecii* [16]. Therefore, serum BDG alone is not specific for diagnosing PJP [17]. However, serum BDG increases only in response to fungi infection [18]. Thus, it can distinguish between *P. jirovecii*-colonization and PJP among PCR-positive patients. However, there are differences in the BDG cut-off values to differentiate *P. jirovecii*-colonization from PJP (33.5 pg/mL by BetaeGlucan test WAKO™ [19], 275 pg/mL by Fungitell assay [20], 200 pg/ mL by Fungitell BDG test [8] and 80 pg/mL by Fungitell kit [21]) due to the heterogeneity of subjects and the differences in BDG detection systems.

This study aimed to establish the cut-off value of serum BDG for diagnosing PJP in HIV-uninfected patients with PCR-positive *P. jirovecii* in the lower respiratory tract. In addition, we further explored the relevant factors affecting the diagnostic performance of BDG, such as age and comorbidities.

## Methods

### Recruitment of participants and study design

This study recruited 330 HIV-uninfected patients with *P. jirovecii* qPCR-positive results from Peking Union Medical College Hospital (PUMCH). Clinical and biological data of all the participants were collected. Only the first qPCR test during hospitalization for patients with multiple *P. jirovecii* qPCR test results was included for subsequent analyses.

Inclusion criteria: (1) HIV-uninfected patients aged 18 to 80 years who were hospitalized in PUMCH from January 1st, 2019, to December 30th, 2021. (2) HIV-uninfected patients with *P. jirovecii* PCR-positive results. The exclusion criteria are listed in the result section.

The gold standard diagnosis of PJP was based on the following criteria: *P. jirovecii* detected using qPCR or microscopy in sputum or BAL specimen; a minimum of two out of three criteria: (1) respiratory symptoms with cough and/or dyspnea, hypoxia; (2) typical radiological picture, including ground glass opacity on a chest computer tomography scan or diffuse interstitial opacity on chest X-ray; (3) receiving an entire course of PJP treatment [22]. Patients with positive *P. jirovecii* qPCR that did not satisfy the above criteria for PJP diagnosis were classified as *P. jirovecii* colonization.

**Fig. 1:**
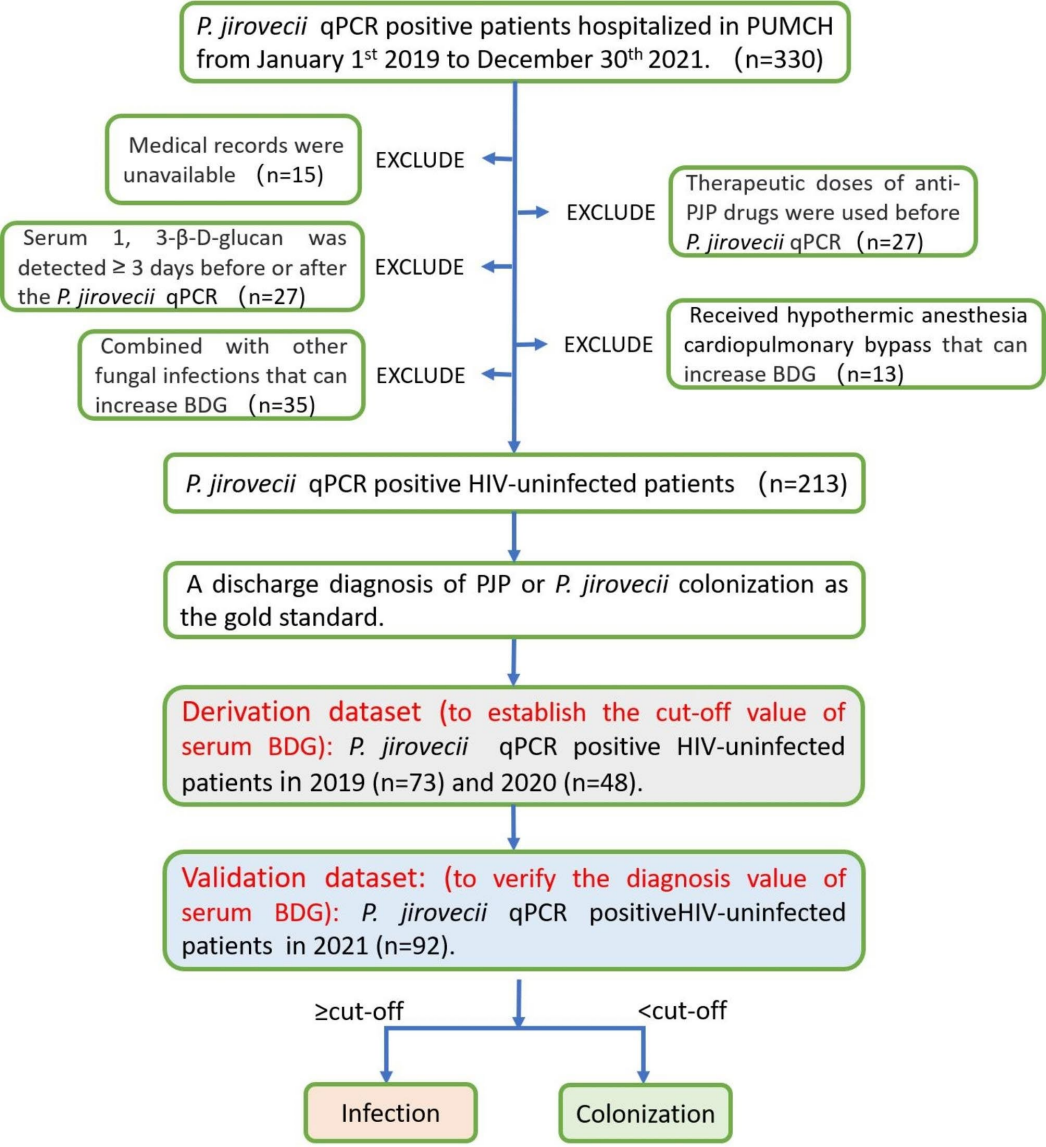
Flow chart of study design. *P. jirovecii* qPCR positive HIV-uninfected patients (n = 330) were recruited from 2019 to 2021. After exclusion, 213 participants were included and divided into a derivation cohort (to define the cut-off value of serum BDG) and a validation cohort (to verify the defined cut-off value of serum BDG). A discharge diagnosis of PJP was taken as the gold standard to divide the participants into two groups: the PJP group and the *P. jirovecii*-colonized group

As shown in Fig. 1, subjects recruited in 2019 (n = 73) and 2020 (n = 48) were pooled and defined as the derivation cohort. Moreover, subjects recruited in 2021 (n = 92) were characterized as the validation cohort. The cut-off value of serum BDG for PJP diagnosis was determined in the derivation cohort and verified within the validation cohort. Sensitivity, specificity, predictive values, ROC curves, and likelihood ratios were used to evaluate the accuracy of the diagnostic model.

### Ethics

The Ethics Committee of Peking Union Medical College Hospital (K22C1279) reviewed and approved the study. This study did not require informed consent for participation following the national legislation and institutional requirements.

### DNA extraction and qPCR of ***P. jirovecii***

Sputum or BALF was collected and mixed with an equal volume of 4% sodium hydroxide solution and incubated at 37 ℃ for 15 min. Then 1mL of the homogenized sample was centrifuged at 12,000 rpm for 5 min, and the pellet was washed using 1mL normal saline. Then 50 μL of 0.5% silica was used for cell wall-broken. The supernatant was extracted through centrifugation after incubating at 100 ℃ for 10 min and used as the template DNA.

The qPCR assay was used to target the mitochondrial gene coding for the large ribosomal subunit (mtLSU) of *P. jirovecii* [23]: mtLSU primers F (5′-CTTAAAATAAATAATCAGACTATGTGCGATAAG-3′) and R (5′-GGAGCTTTAATTACTGTTCTGGGC-3′); with Taq probe 5′-FAM-TAGATAGTCGAAAGGGAA-MGB-3′. Additionally, the primers and probes targeting the human album gene became the internal controls: primers F (5′-GCTGTCAT CTCTTGTGGGCTG-3′) and R (5′-ACTCATGGGAGCTGCTGGTTC-3′); with Taq probe 5′-VIC-GGAGAGATTTGTGTGGGCATGACA-TRAMA − 3′.

Each TaqMan reaction mix had 15 μL of 2× PCR master mix (Promega, Beijing, China), 1 μL forward primers, 1 μL reverse primers, and 1 μL probe for both mtLSU and album. It also had 3 μL of DNA template and 1 μL of ddH_2_O to make the final volume of 25 μL. Reactions were performed using ABI 7500 (Applied Biosystems, Foster City, CA, USA). The reaction conditions were: 95 ℃ for 5 min, followed by 40 cycles of denaturation at 95 ℃ for 3s and finally annealing/extension at 60 ℃ for 30s.

The result was valid only when the positive (PC) and negative (NC) controls were regulated: the cycle threshold (Ct) value of PC ≤ 37, the Ct value of NC was 0, and the Ct value of the album gene was between 20 and 39 in all the tested samples. The result was considered positive when the Ct value of the mtLSU gene ≤ 37 and negative when Ct > 37.

### BDG tests

Fresh serum samples were analyzed for BDG using the Fungus (1–3)-ß-D Dextran Test Kit (Zhanjiang A&C, Guangdong, China). The sera were pretreated and analyzed based on the manufacturer’s instructions. In brief, serum was diluted 10-fold with sample diluent after centrifugation and incubated at 75℃ for 10 min. Then 100 μL pretreated sample was mixed using 50 μL reagent solution in a reaction tank and monitored using an LKM Kinetic Tube Reader (Lab Kinetics, UK) for 60 min.

The maximum detection limit was 10,000 pg/mL, and BDG levels below 10 pg/mL (the minimum detection limit) were set at 10 pg/mL. BDG values ≤ 100.5 pg/mL and ≥ 151.5 pg/mL were considered negative and positive, respectively, and between 100.5 and 151.5 pg/mL was considered indeterminate. Certified laboratory technicians from the Department of Clinical Laboratory at PUMCH duplicated all the analyses.

### Statistics

Fisher exact test was used for the analysis of gender. *χ*^*2*^ test was used for the analysis of comorbid composition. Mann-Whitney test was used to analyze age, percentages, and counts of lymphocytes, CD4^+^T cell to CD8^+^T cell ratio, C reactive protein (CRP), and erythrocyte sedimentation rate (ESR). The receiver operator characteristic curve (ROC) was used to evaluate the diagnostic value of serum BDG in both the derivation and validation cohorts. Differences were considered significant at *P* < 0.05. All the statistical analyses were performed with Prism 9.3.1 (GraphPad Software).

## Results

### Description of the study population

We excluded 117 cases from this study. Firstly [24, 25], therapeutic doses of sulfonamides would affect serum BDG and *P. jirovecii* qPCR tests. Thus, 27 such cases were excluded. Secondly, 27 cases exceeding a long interval (> 3 days) that would affect the consistency between serum BDG and *P. jirovecii* qPCR [25] were excluded. Thirdly, co-infection with other fungi [26] would also elevate serum BDG, leaving the contribution of PJP uncertain; thus, 35 such cases were excluded. Fourthly, we found that cardiopulmonary bypass could lead to a spurious increase in BDG (data unpublished), so 13 cases were excluded. Finally, 15 cases with incomplete medical records were excluded.

Ultimately, there were 213 patients enrolled in the study (Fig. 1), 51.6% were female, and their median age was 60 years. There were 22 (10.3%) with hematologic malignancies, 31 (14.6%) with solid cancer, 100 (46.9%) with autoimmune or inflammatory disorders, 15 (7.0%) with interstitial pneumonia due to noninfectious causes, 24 (11.3%) with nephrotic syndrome, 14 (6.6%) with infectious diseases (severe infections caused by bacteria and/or viruses), and 7 (3.3%) with other diseases (including 4 thrombotic diseases and 3 pericarditis). Based on the gold standard, there were 159 PJP patients and 54 *P. jirovecii*-colonized patients.

### Differences in mortality and comorbid composition between PJP and ***P. jirovecii***-colonized patients

Based on the gold standard, there were 159 PJP patients and 54 *P. jirovecii*-colonized patients. The colonization rate was 25.4%, consistent with other studies (24.5%) [8]. As indicated in Table 1, no significant differences existed in age (*P* = 0.1803) and gender (*P* = 0.0827) between the two groups. In contrast, the comorbid composition was significantly different (*P* = 0.0227). The in-hospital mortality was significantly higher in the PJP group than in the *P. jirovecii*-colonization group (13.2 vs. 3.7%, *P* = 0.0374), consistent with previous studies [1, 3]. Furthermore, the proportion of BALF samples in the PJP group was significantly higher than that in the *P. jirovecii*-colonization group (37.7% vs. 9.3%, *P* < 0.0001). This is because patients with PJP are more likely to meet the indication for alveolar lavage operation than patients with *P. jirovecii*-colonization.

**Table 1:** Characteristics of the investigated population

	PJP (n = 159)	*P. jirovecii* colonization (n = 54)	*P* value
**Age (years)**	59(44, 67)	62.5(48,70)	**0.1803**
**Women (n=, %)**	88(55.3)	22(40.7)	**0.0827**
**Comorbid conditions**			**0.0227**
Hematologic malignancies (n=, %)	18 (11.3)	4 (7.4)	
Solid cancer (n=, %)	20 (12.6)	11 (20.4)	
Autoimmune or inflammatory disorders (n=, %)	77 (48.4)	23 (42.6)	
Interstitial pneumonia (n=, %)	12 (7.5)	3 (5.6)	
Nephrotic syndrome (n=, %)	22 (13.8) *	2 (3.7)	
Infectious diseases (n=, %)	7 (4.4)	7 (13.0)	
Other diseases (n=, %)	3 (1.9)	4 (7.4)	
**In-hospital mortality rates (n=, %)**	21 (13.2)	2 (3.7)	**0.0374** ^**#**^
**Sample types**			
Sputum (n=, %)	99 (62.3)	49 (90.7)	**< 0.0001**
Bronchoalveolar lavage fluid (n=, %)	60 (37.7)	5 (9.3)	
**Serum BDG (pg/mL)**	363.4 (174.7, 928.8)	26.45 (12.58, 61.25)	**< 0.0001**

Mann-Whitney test was used for analysis of age and serum BDG. Fisher’s exact test was used for analysis of gender and sample types. Chi-square test was used for analysis of comorbid composition and in-hospital mortality rates (one-sided test). * The proportion of nephrotic syndrome was compared with the proportion of the combination of other comorbid between the two groups (*P* = 0.0465). ^#^ One sided *P* value. PJP: *Pneumocystis jirovecii* (formerly *Pneumocystis carinii*) pneumonia

### Differences in lymphocytes and inflammatory factors between PJP and ***P. jirovecii***-colonized patients

**Fig. 2:**
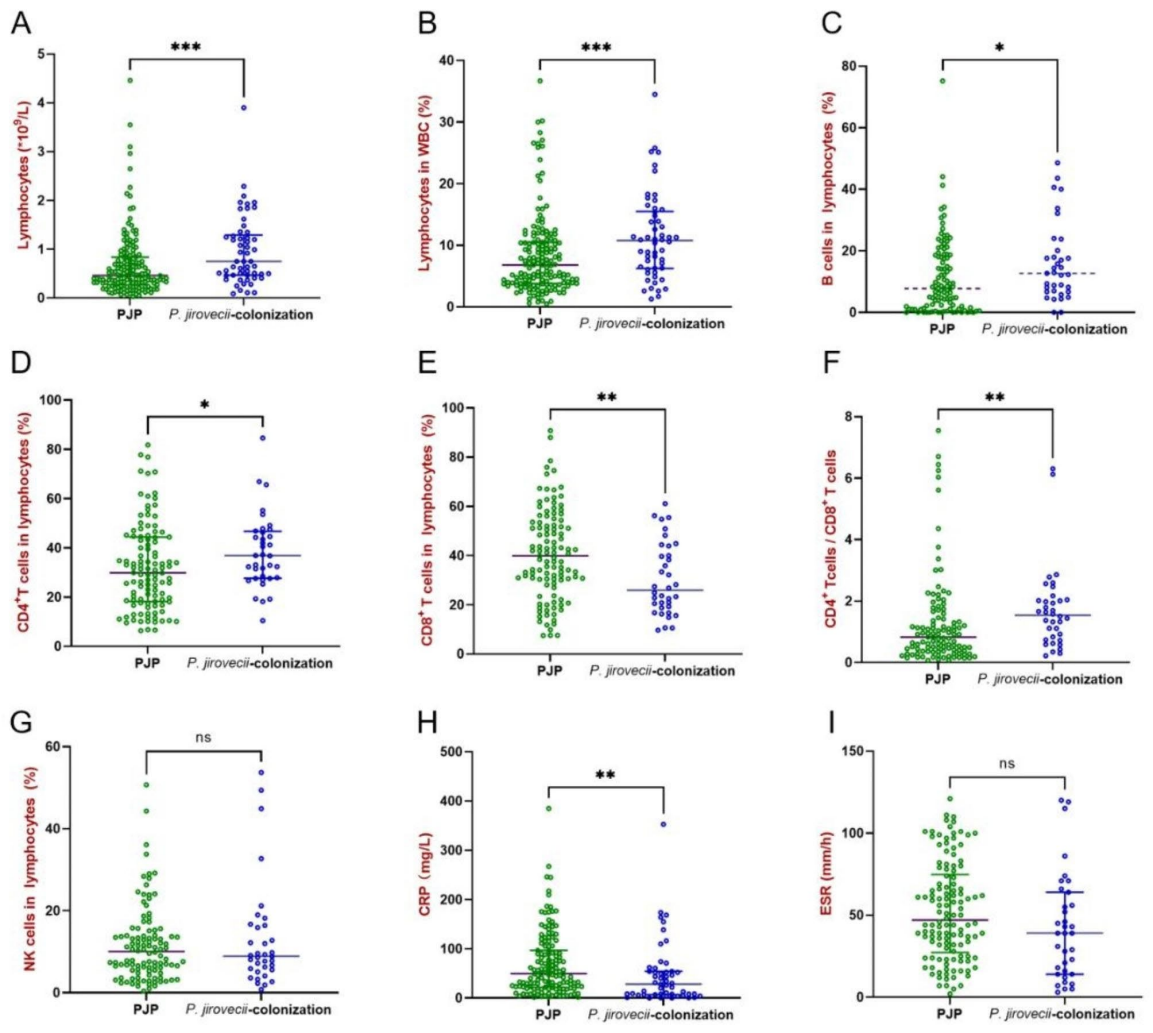
Peripheral lymphocytes and inflammatory indicators were compared between PJP and *P. jirovecii*-colonized patients. Differences of lymphocyte counts (**A**), lymphocyte percentage (**B**), B cell percentage (**C**), CD4^+^T cell percentage (**D**), CD8^+^T cell percentage (**E**), CD4^+^T cell to CD8^+^T cell ratio (**F**), NK cell percentage (**G**), C reactive protein (**H**), and erythrocyte sedimentation rate (**I**) between PJP and *P. jirovecii*-colonized patients. The *p*-values were calculated using Mann-Whitney tests, and statistical significance is displayed as *P < 0.05, **P < 0.01, ***P < 0.001

As shown in Fig. 2, both lymphocytic counts and proportions were significantly lower in PJP patients (0.46 × 10^9^/L, 6.8%) than in *P. jirovecii*-colonized patients (0.75 × 10^9^/L, 10.8%; *P* = 0.0004, *P* = 0.0009), being consistent with other studies [27]. Additionally, the percentage of B cells in lymphocytes was reduced in PJP patients (7.8% vs. 12.7%; *P* = 0.0187). In contrast, the percentage of NK cells in lymphocytes was comparable between the two groups (10.1% vs. 8.9%; *P* = 0.9953).

Notably, CD4^+^ T cells and CD8^+^ T cells revealed an opposite trend. The percentage of CD4^+^ T cells in lymphocytes decreased among PJP patients (29.9% vs. 36.9%; *P* = 0.0265), consistent with previous studies [28, 29]. In contrast, the percentage of CD8^+^ T cells in lymphocytes increased among PJP patients (39.9% vs. 25.9%; *P* = 0.0030), inconsistent with previous studies [30]. Consequently, the ratios of CD4^+^ T cells to CD8^+^ T cells were lower among PJP patients (0.8 vs. 1.5; *P* = 0.0015), indicating that PJP patients have both immunodeficiency and hyperimmunity.

Moreover, two inflammatory factors, CRP and ESR, were higher in PJP patients (49.6 mg/L, 47 mm/h) than in *P. jirovecii*-colonized patients (27.9 mg/L, 39 mm/h). However, a significant difference was only observed in CRP (*P* = 0.0020), reflecting the hyperinflammatory state correlated with *P. jirovecii* infection.

### ROC curve and cut-off value of serum BDG for the diagnosis of PJP

**Fig. 3:**
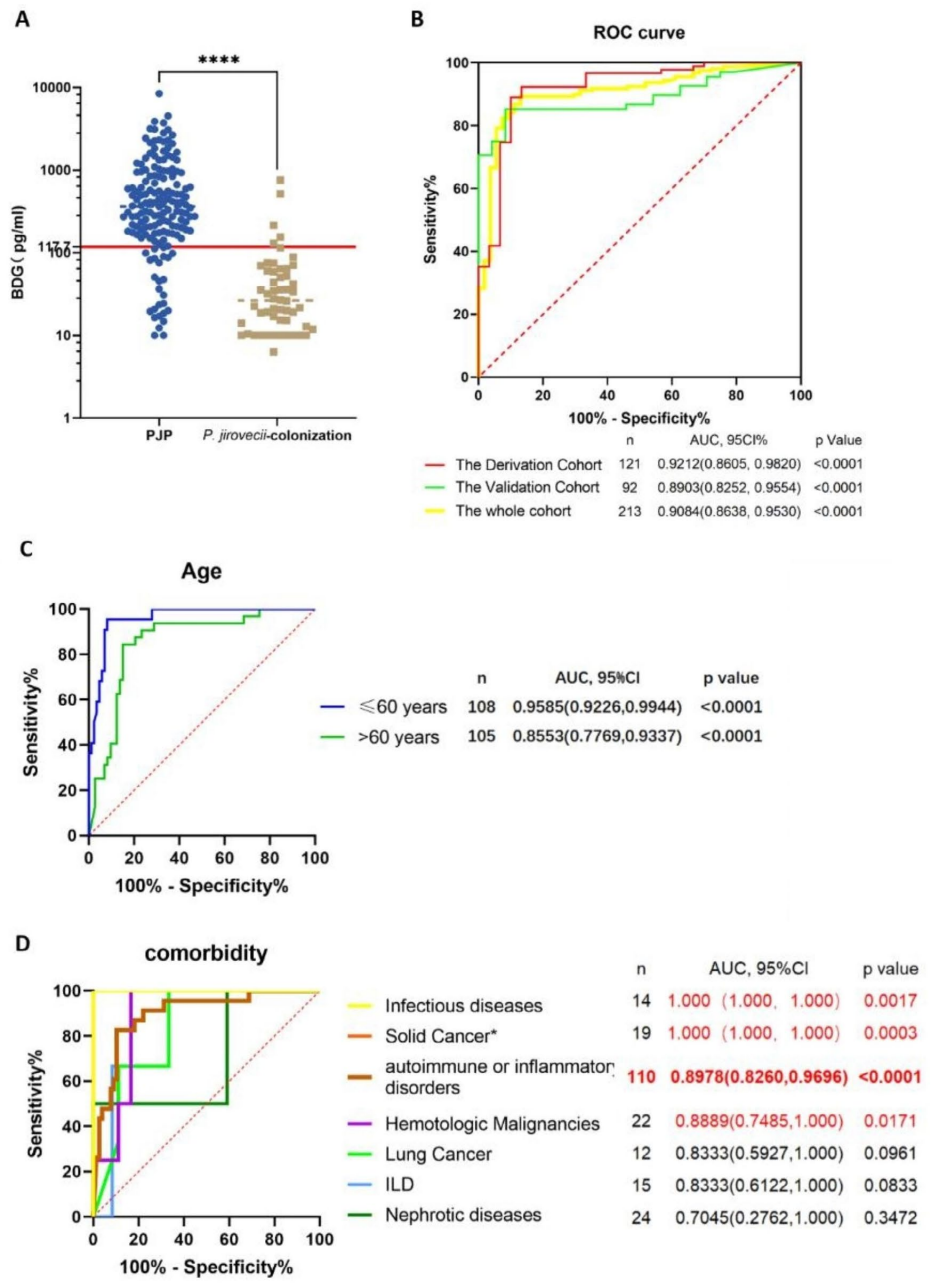
ROC curves of serum BDG in different cohorts and subgroups. (**A**) Comparison of serum BDG between PJP and *P. jirovecii-*colonized patients. (**B**) ROC curves of serum BDG based on the Derivation cohort, Validation cohort and the total cohort. (**C**) ROC curves of serum BDG based on subjects aged ≤ 60 years and > 60 years. (**D**) ROC curves of serum BDG based on subjects with comorbidities of infectious diseases, solid cancer, autoimmune or inflammatory disorders, hematologic malignancies, lung cancer, ILD, or nephrotic diseases. * Patients with lung cancer were excluded from the solid tumor subgroup. The *p*-values were calculated using Mann-Whitney test, and statistical significance is displayed as ****P < 0.0001

As indicated in Table 1; Fig. 3A, the serum BDG level of PJP patients was significantly higher than those in *P. jirovecii*-colonized patients (*P* < 0.0001). The AUC of derivation cohort, validation cohort, and total subjects were 0.92, 95% CI (0.86, 0.98); 0.89, 95% CI (0.82, 0.95); and 0.91, 95% CI (0.86, 0.95); respectively (Fig. 3B).

A serum BDG cut-off of 117.7 pg/mL was determined using the derivation cohort and then verified by the validation cohort. As shown in Table 2, with the prevalence of 74.6%, BDG ≥ 117.7 pg/mL showed outstanding specificity, LR, and PPV in the derivation (90.0%, 8.9, and 96.4%) and validation (91.7%, 9.2, and 96.3%) cohort. At the same time, the sensitivity and NPV were slightly lower in the derivation cohort (89.0% and 73.0%) and lowered in the validation cohort (76.5% and 57.9%). Notably, the median value of serum BDG in the derivation cohort was significantly higher than in the validation cohort (300.3 pg/mL vs. 170.2 pg/mL, *P* = 0.0253). This could be related to its better diagnostic efficacy in the derivation cohort than in the validation cohort.

**Table 2:** Evaluation of diagnostic performance of serum BDG (at ≥ 117.7 pg/mL)

Subgroups	Sen (%)	Spe (%)	LR	NPV (%)	PPV (%)
Derivation cohort (n = 121)	89.01	90.00	8.900	72.97	96.43
Validation cohort (n = 92)	76.47	91.67	9.176	57.89	96.30
≤ 60 years (n = 108)	89.53	95.45	19.70	70.00	78.57
> 60 years (n = 105)	78.08	87.50	6.247	63.63	93.44
Hematologic malignancies (n = 22) *	83.33	100.0	3.333	57.14	100.0
Solid cancer (n = 31)	80.00	100.0	9.350	73.33	100.0
Autoimmune or inflammatory disorders (n = 100)	83.12	82.61	4.779	61.29	94.20
Interstitial pneumonia (n = 15) *	66.67	100.0	2.000	42.86	100.0
Nephrotic syndrome (n = 24) *	100.0	50.00	2.000	100.0	95.65
Infectious diseases (n = 14)	100.0	100.0	7.000	100.0	100.0
Lung cancer (n = 12) *	55.56	100.0	2.000	42.86	100.0

Sen: sensitivity; Spe: specificity; LR: likelihood ratio; NPV: negative predictive value; PPV: positive predictive value. The * indicates that the number of *P. jirovecii*-colonized cases was < 5

### Age and comorbidities affect the diagnostic efficacy of serum BDG

Stratified ROC analysis was performed based on comorbidities (Table 2). As indicated in Fig. 3D, the AUC of serum BDG was 1.00 in the infectious disease subgroup, 0.90 in the solid cancer subgroup, and 0.89 in the autoimmune or inflammatory disorders subgroup, 0.89 in the hematologic malignancy subgroup, 0.83 in both the lung cancer and the ILD subgroups, and 0.70 in the nephrotic syndrome subgroup. Therefore, the diagnostic efficacy of BDG varied in patients with varied comorbidities, though without statistical difference (*P* = 0.3698). BDG could not significantly distinguish PJP from *P. jirovecii*-colonization in subgroups of lung cancer, ILD and nephrotic syndrome (*P* = 0.0961, *P* = 0.0833, and *P* = 0.3472). Of note, the suboptimal AUC could also be related to the limited number of *P. jirovecii*-colonized cases (n < 5) in the subgroups (Table 2; Fig. 3).

Moreover, stratified ROC analysis was performed by dividing the subjects into two subgroups: ≤ 60 years (n = 108) and > 60 years groups (n = 105). As represented in Fig. 3C, the AUC of serum BDG was higher in patients aged ≤ 60 years (0.96) than in patients aged > 60 years (0.86), but the difference was not statistically significant (*P* = 0.1196).

As shown in Table S1, PJP patients with lung cancers had the lowest median BDG levels(151.6), although the difference was not statistically significant (*P* = 0.1971).

### Preliminary analysis of false-negative related factors

The sensitivity and NPV of BDG were suboptimal, particularly in the validation cohort. We compared the characteristics of the true-positive group (n = 137) and the false-negative group (n = 22) to analyze the factors associated with false negatives (Table 3).

**Fig. 4:**
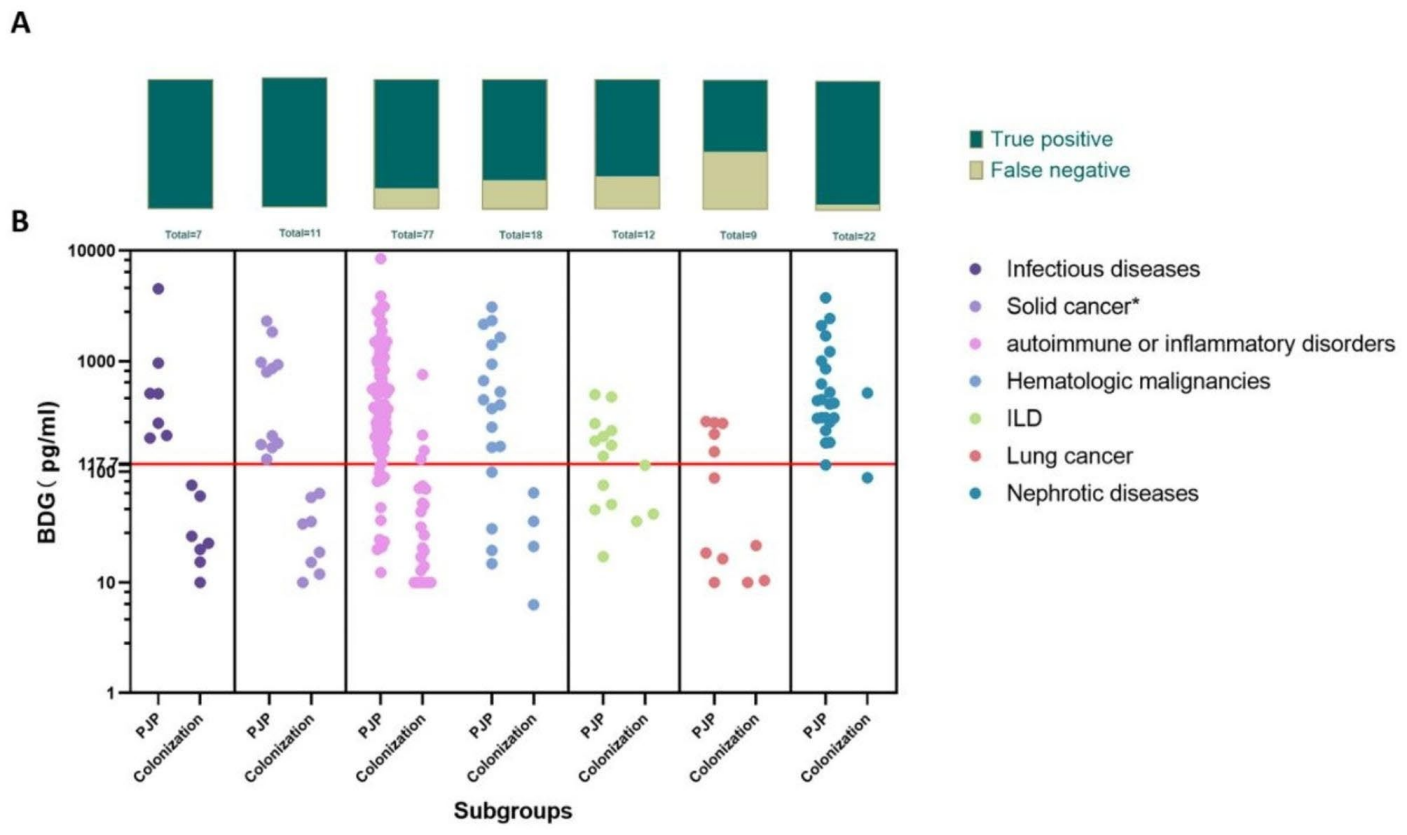
Distribution of BDG in the PJP and *P. jirovecii-*colonized groups. (**A**) In each subgroup, PJP patients were diagnosed by BDG ≥ 117.7pg/mL, proportions of true positive and false negative cases were shown in proportion composition diagrams. (**B**) Scatter plot showing comparison of serum BDG between PJP and *P. jirovecii-*colonized patients in different subgroups. * Patients with lung cancer were excluded from the solid tumor subgroup

In the current study, there were 3,4,12,and 3 PJP cases not identified by BDG in the hematologic malignancie, lung cancer, autoimmune or inflammatory disorders, and ILD subgroups, respectively (Fig. 4). And the proportion of lung cancer and ILD in the false-negative group (18.2%, 13.6%) was higher than that in the true-positive group (6.3%, 9.5%), though without a statistical difference (*P* = 0.2815). Furthermore, there were no significant differences in peripheral lymphocyte count, peripheral lymphocyte proportion and gender composition between the true positive group and the false negative group (*P* = 0.4353, *P* = 0.1422, and *P* = 0.1059). Of interest, the median age of the false negative group was significantly higher than that of the true positive group (63 vs. 58 years, *P* = 0.0259) (Table 3). OR analysis showed that when the patient was older than 57 years old, the odds ratio of false negative to true positive were 4.307 (p = 0.007).

**Table 3:** Comparison of characteristics between true positive group and false negative group

	True positive(n = 137)	False negative (n = 22)	*P* value
**Age (years)**	58 (41.5, 67)	63 (58, 68.5)	**0.0259**
**Women (n=, %)**	79(56.8)	8(36.4)	**0.1059**
**Lymphocytes (%)**	7 (3.9, 11.0)	4.9 (3.5, 8.8)	**0.1422**
Lymphocytes (*10 ^**9**^ **/L)**	0.46 (0.26, 0.91)	0.44(0.29, 0.69)	**0.4353**
**Comorbid conditions**			**0.2815**
Hematologic malignancies (n=, %)	15 (15.8)	3 (13.6)	
Lung cancer	6 (6.3)	4 (18.2)	
Autoimmune or inflammatory disorders (n=, %)	65 (68.4)	12 (54.5)	
Interstitial pneumonia (n=, %)	9 (9.5)	3 (13.6)	

Mann-Whitney test was used for analysis of age, counts and proportions of lymphocytes. Fisher’s exact test was used for analysis of gender and Chi-square test was used for analysis of comorbid composition

### Odds ratios for PJP versus ***P. jirovecii***-colonization was correlated with immune markers, inflammatory markers, and history of glucocorticoid therapy

**Fig. 5:**
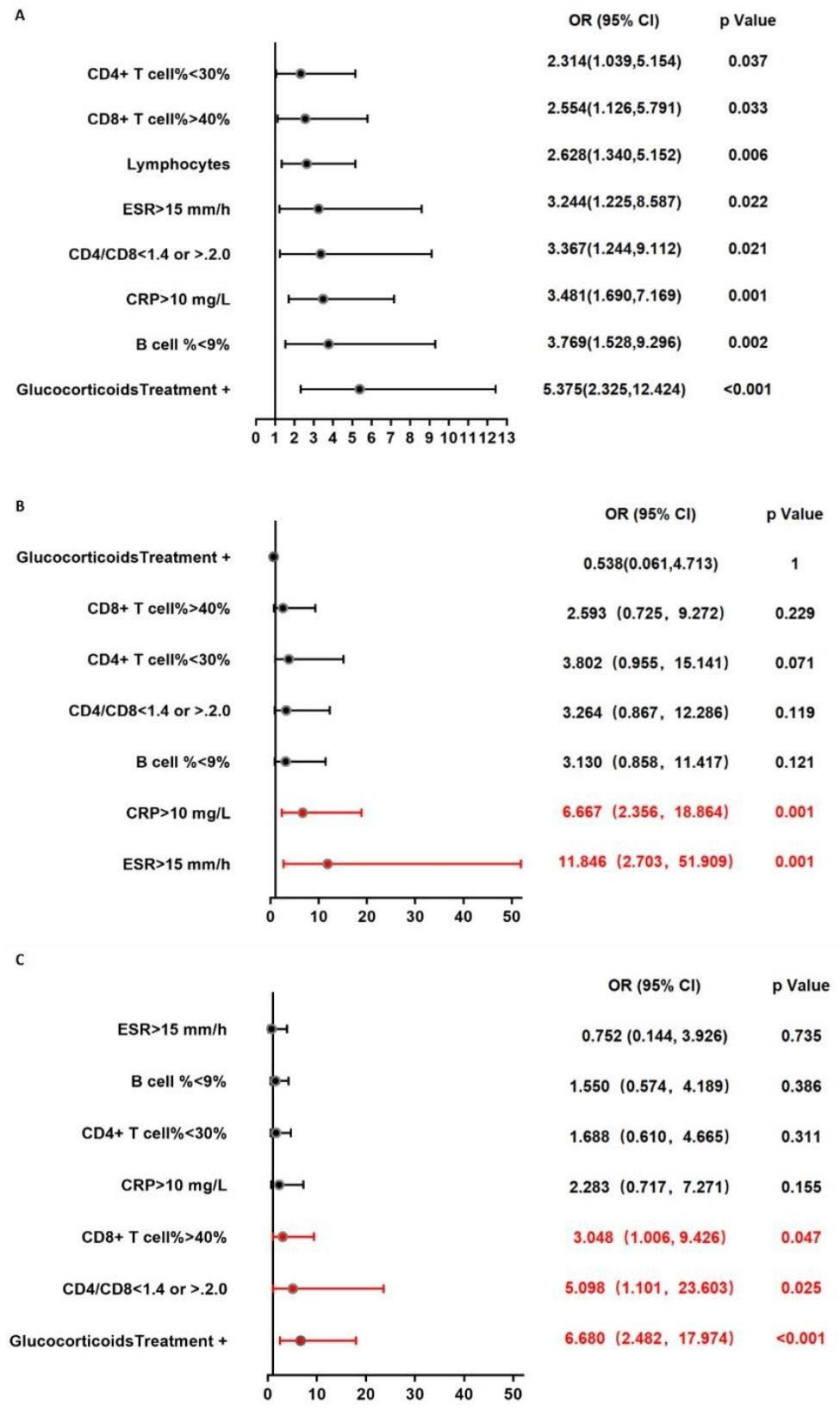
Odds ratios of PJP versus *P. jirovecii-*colonization with different characteristics in the whole cohort and Autoimmune or inflammatory disorders subgroup. (**A**) In the whole cohort, Odds ratios of PJP versus *P. jirovecii-*colonization elevated significantly when proportion of CD4^+^ T cells in T cells < 30%, proportion of CD8^+^ T cells in T cells > 40%, lymphocyte counts < 1.0×10^9^/L, ESR > 15 mm/h, CD4^+^T/ CD8^+^T < 1.4 or > 2.0, CRP > 10 mg/L, proportion of B cells in lymphocytes < 9% and treatment with glucocorticoids within 2 weeks. From top to bottom, the odds ratios are arranged from small to large. (**B**) In the autoimmune or inflammatory disorders subgroup, odds ratios of PJP versus *P. jirovecii-*colonization elevated magnificently when ESR > 15 mm/h, CRP > 10 mg/L. (**C**) For subjects without autoimmune or inflammatory disorders, odds ratios of PJP versus *P. jirovecii-*colonization elevated magnificently when CD8^+^ T cells in T cells > 40%, CD4^+^T/ CD8^+^T < 1.4 or > 2.0, and treatment with glucocorticoids within 2 weeks. The *p*-values were calculated using Chi-square test and Fisher test

As shown in Fig. 5, for the whole subjects, the odds ratios for PJP versus *P. jirovecii*-colonization were 2.314, 2.554, 2.628, 3.244, 3.367, 3.481, 3.769, and 5.375, respectively, when the proportion of CD4^+^ T cells in T cells < 30%, the proportion of CD8^+^ T cells in T cells > 40%, lymphocyte counts < 1.0×10^9^/L, ESR > 15 mm/h, CD4^+^T/CD8^+^T < 1.4 or > 2.0, CRP > 10 mg/L, the proportion of B cells in lymphocytes < 9% and had a history of treatment with glucocorticoids within 2 weeks. For patients with autoimmune or inflammatory disorders, the odds ratios for PJP versus *P. jirovecii*-colonization were 6.667 and 11.846 when CRP > 10 mg/L and ESR > 15 mm/h. For participants without autoimmune or inflammatory disorders, odds ratios for PJP versus *P. jirovecii*-colonization were 3.048, 5.098 and 6.680 when proportion of CD8^+^ T cells in T cells > 40%, CD4^+^T/CD8^+^T < 1.4 or > 2.0, and had a history of treatment with glucocorticoids within 2 weeks.

## Discussion

A timely and accurate PJP diagnosis can reduce PJP-related mortality [31–34]. However, the clinical and imaging findings are poorly specific. The highly sensitive *P. jirovecii* qPCR assay cannot distinguish PJP from *P. jirovecii*-colonization [35]. Fortunately, although serum BDG lacks specificity for the diagnosis of PJP [20], its elevation is primarily a response to fungal infection rather than colonization [18, 36, 37]. Herein, we evaluated the diagnostic potential of serum BDG for PJP in HIV-uninfected patients who were PCR-positive for *P. jirovecii*.

We had 213 subjects: 159 PJP and 54 *P. jirovecii*-colonized patients, with PJP patients having higher mortality. It was also observed that lymphocytes, B cells, and the ratio of CD4^+^T cells to CD8^+^T cells were significantly decreased in PJP patients, while CD8^+^T cells and CRP were significantly increased, suggesting that PJP is related to host immune statu [28, 29]. Similarly, the odds ratio for PJP versus *P. jirovecii*-colonization was significantly elevated when the percentage of CD4^+^ T cells < 30%, CD8^+^ T cells > 40%, lymphocyte counts < 1.0×10^9^/L, ESR > 15 mm/h, CD4^+^T/CD8^+^T < 1.4 or > 2.0, CRP > 10 mg/L, B cells < 9% of lymphocytes, and a history of treatment with glucocorticoids within 2 weeks. Of note, glucocorticoid treatment significantly increased the risk of PJP in non-CTD patients, consistent with previous studies that identified prolonged systemic corticosteroid therapy as an imporatnt risk factor for the development of PJP [38]. For subjects without autoimmune or inflammatory disorders, abnormality of CD4^+^T/CD8^+^T (< 1.4 or > 2.0) and increased CD8^+^ T cell percentages (> 40%) can significantly increase the risk of PJP. Animal models have shown that the inflammatory response of CD8^+^T cells to *P. jirovecii* directly impairs lung function and is involved in the pathogenesis of PJP [39]. For patients with autoimmune or inflammatory disorders, elevated inflammatory markers, such as CRP (> 10 mg/L) and ESR (> 15 mm/h), suggest an increased risk of PJP. Elevated CRP was found to be associated with low PaO_2_ and poor prognosis in HIV-positive PJP patients [40].

Regarding the diagnostic value of serum BDG, at ≥ 117.7 pg/mL, the specificity and PPV were outstanding, while the sensitivity and NPV were slightly insufficient (89.0 and 76.5%). Similarly, previous studies have shown an overall sensitivity of 83 ~ 89% for the diagnosis of PJP based on BDG alone in HIV-negative patients [17, 39–41]. Some experts have suggested that BDG testing should not be used alone to rule out PJP in immunocompromised pneumonia patients [17, 42]. Some studies reported a higher NPV in BDG, which may be related to a lower PJP prevalence (55.9% [19], 22.0% [20], or 21.4% [21] ). A study [8] with a similar PJP prevalence (75.5%) as ours reported a similar BDG NPV (52.0%). Therefore, it is necessary to be aware of the low NPV of BDG when PJP is excluded solely based on negative BDG for HIV-uninfected patients with *P. jirovecii* PCR-positive.

Furthermore, stratified ROC analysis suggested that the diagnostic efficacy of BDG varied in different age subgroups and different comorbidities. This study preliminarily found that BDG ≥ 117.7 pg/mL has insufficient diagnostic dfficacy for PJP in patients with lung cancer, ILD and nephrotic syndrome. We speculate that in patients with lung cancer and ILD, regional or diffuse parenchymal blood supply insufficiency may contribute to decreased BDG levels. Of note, PJP patients with lung cancers had the lowest median BDG levels, though without statistical difference. We speculate that the immune cells in the local lung tissue of lung cancer are in an immunosuppressive state, which may also lead to insufficient BDG. In patients with nephrotic syndrome, continuous renal replacement therapy, dialysis and plasma exchange, may contribute to false negative or positive BDG levels. It should be noted that due to the small sample size of colonized patients (n < 5) in the above-mentioned comorbidities, which may introduce bias and needs to be further confirmed by larger sample size studies.

We initially explored the factors influencing the diagnosis of PJP by BDG. Aging was characterized as false-negative related factors. With the increase of age, the diagnostic efficacy decreases, and the mechanism needs to be further studied. In this study, although lymphocytes, B cells, and CD4^+^T cells in PJP patients were significantly lower than those in *P. jirovecii* colonized patients, the number and proportion of peripheral blood lymphocytes did not affect the diagnostic efficacy of serum BDG.

Other than patient factors, the BDG detection process also impacts the diagnostic performance of BDG. The measurement of serum BDG is based on the Limulus test [26], which could be affected by many factors. False-positive related factors include lentinan intake [41], intravenous injection with immunoglobulins, albumin, coagulation factors [43] or antibiotics such as amoxicillin/clavulanic acid [44], hemodialysis using cellulose-containing filters [45], and contact with surgical sponges or gauze [46]. There are also factors associated with false negatives: Hyperpigmented serums (bilirubin or triglycerides) [47] and antifungal treatment [48]. In the current study, most of these interference factors were avoided during and after serum BDG detection.

Our study identified an ideal cut-off value of ≥ 117.7 pg/mL. It was different from the cut-off values (including 33.5 pg/mL [19], 275 pg/mL [20], 200 pg/mL [8], or 80 pg/mL [21]) provided by other studies. This may be related to using different BDG detection kits: most studies used Fungitell [8, 20, 21], whose reagents were derived from *Limulus polyphemus*. In contrast, we used Fungus (1–3)-ß-D Dextran Test Kit, with reagents derived from *Tachypleus tridentatus*. The cut-off values of different detection systems are not comparable. Therefore, it is necessary to establish the cut-off values separately.

The current retrospective study has certain limitations. First, a relatively small number of samples could lead to a computational performance bias. Future studies with larger sample sizes should verify the conclusion drawn from the stratified analysis in this study. Another limitation is that PJP patients with other fungal infections, which are not clinically uncommon, were excluded from this study. Therefore, whether the cut-off value from this study also applies to the diagnosis of PJP in patients with co-infection having other fungal infections needs further investigation.

## Conclusion

To sum up, the diagnostic efficacy of BDG is affected by comorbidities and age. Serum BDG ≥ 117.7 pg/mL could effectively distinguish *P. jirovecii*-colonization from infection in qPCR-positive HIV-uninfected patients with infectious diseases, solid tumors (excluding lung cancer), autoimmune or inflammatory disorders, and hematological malignancies, which is helpful for the timely initiation of anti-PJP therapy. Of note, for patients with lung cancer, ILD, and nephrotic diseases, PJP should be cautiously excluded at BDG < 117.7 pg/mL. Furthermore, history of glucocorticoid treatment, abnormal T cell percentage, elevated CRP and ESR are risk factors for PJP.

### Electronic supplementary material

Below is the link to the electronic supplementary material.

Supplementary Material 1

## Data Availability

The original data in this study are available upon reasonable request from the corresponding author.
