# Supplementary material for: Negative serum (1,3) -β-D-glucan has a low power to exclude Pneumocystis jirovecii pneumonia (PJP) in HIV-uninfected patients with positive qPCR
Source: Ann Clin Microbiol Antimicrob. 2023 Nov 20;22:102. doi: 10.1186/s12941-023-00650-7 (PMC10662630; doi:10.1186/s12941-023-00650-7)
Supplement: Supplementary file 1 — Supplementary Material 1 [file 12941_2023_650_MOESM1_ESM.docx]

**Table S1** Serum BDG median of PJP and *P. jirovecii*-colonized patients in different subsets.

| **Serum BDG (pg/mL)** | **PJP** | ***P. jirovecii-*colonization** | **AUC** |
| --- | --- | --- | --- |
| **Age** |  |  |  |
| **≤ 60 years** | 444.6 (n=86) | 19.3 (n=22) | 0.9585 |
| **>60 years** | 274.5 (n=73) | 29.8（n=32） | 0.8553 |
| **Comorbid conditions** |  |  |  |
| Hematologic malignancies (n=22) | 426.0 (n=18) | 28.4 (n=4) | 0.8889 |
| Solid cancer (n=31) | 215.9 (n=20) | 18.7 (n=11) | 0.9000 |
| Autoimmune or inflammatory disorders (n=100) | 381.8 (n=77) | 26.8 (n=23) | 0.8978 |
| Interstitial pneumonia (n=15) | 181.6 (n=12) | 41.6 (n=3) | 0.8333 |
| Nephrotic syndrome (n=24) | 429.3 (n=22) | 302.2 (n=2) | 0.7045 |
| Infectious diseases (n=14) | 460.1 (n=7) | 22.6 (n=7) | 1.000 |
| Lund cancer (n=12) | 151.6 (n=9) | 10.4 (n=3) | 0.8333 |
